# Irrigation of the Nasal Passages

**Published:** 1866-09

**Authors:** H. A. Johnson

**Affiliations:** 611 Wabash Avenue; Chicago


### IRRIGATION OF THE NASAL PASSAGES.

By H. A. JOHNSON, M.D., Chicago.

The readers of the EXAMINER are, no doubt, aware that the nasal passages may be irrigated, by allowing a stream of water to run into one nostril, passing back behind the septum and issuing from the other nostril, the subject in the meantime breathing through the open mouth; and that by this means medicinal agents may be applied with ease to parts that are reached with difficulty by other devices.

TIEMAN, of New York, has manufactured an instrument especially for the purpose, but its cost is considerable, and for some months I have been supplying my patients with a very simple and cheap contrivance, meeting perfectly the objects in view. It consists of a gum elastic tube three feet in length, to one end of which is attached a hard rubber nozzle, such as is ordinarily supplied to the hard rubber syringe; to the other, a glass tube bent so as to hang over and down into a common water pitcher. This tube is used as a syphon. The pitcher, containing the solution to be used, is placed about two feet above the head of the patient, and, the tube having been first filled with water, the bent portion is placed in the solution, and, holding the other extremity between the thumb and finger so as to control the current, the nozzle is placed in one nostril, and, the pressure being removed, the solution passes through the nares and issues from the other nostril.

In chronic disease of the passages, and especially where they become filled with accumulations of dried mucus, I have obtained excellent results from this treatment.

Solutions of chlorate of potash, chloride of zinc, bromide of ammonium, etc., may be used and in large quantities.

The first effect is usually mechanical, the removal of the accumulated matters. When this is accomplished, the medicinal effect of remedies addressed to the vital properties of the tissues will be more certainly realized.

*611 Wabash Avenue, Aug. 15, 1866.*

